# Impact of Rotten Eggs on Hatchery Performances: A Multicentric Study

**DOI:** 10.3390/ani10101725

**Published:** 2020-09-23

**Authors:** Giovanni Franzo, Wessel Swart, Miren Arbe Ugalde, Higor Cotta, Mathilde Lecoupeur, William Boyer, Kostas Koutoulis, Mattia Cecchinato

**Affiliations:** 1 Department of Animal Medicine, Production and Health (MAPS), University of Padua, 35020 Legnaro, Italy; mattia.cecchinato@unipd.it; 2 Ceva Santé Animale, 33500 Libourne, France; wessel.swart@ceva.com (W.S.); miren.arbe@ceva.com (M.A.U.); higor.cotta@ceva.com (H.C.); mathilde.lecoupeur@ceva.com (M.L.); william.boyer@ceva.com (W.B.); 3 Department of Poultry Diseases, Faculty of Veterinary Medicine, University of Thessaly, 43100 Karditsa, Greece; kkoutoulis@vet.uth.gr

**Keywords:** hatchery, rotten eggs, performances, multicentric, chickens

## Abstract

**Simple Summary:**

Improving day-old chick quality is essential for the overall profitability of the broiler productive cycle and has been associated with a decreased feed conversion rate, increased growth performance, resistance to infectious diseases, and welfare parameters. The hatchery practices are fundamental, since adequate hygiene and handling are crucial in reducing egg contamination and cross-contamination. Particularly, the efficient removal of rotten eggs has been suggested to reduce the overall bacterial burden contaminating the needle used for in-ovo vaccination, the nearby eggs, and the whole incubator/hatching room when broken. In the present multicentric study, including 11 European countries, a remarkable impact of the rotten egg percentage on hatchery productive parameters, such as the hatchability, embryo mortality, and level of contamination, was demonstrated. Efficient rotten egg removal and the application of new generation technologies for appropriate detection and removal tools should thus provide remarkable benefits for hatchery performances and indirectly for downstream poultry production.

**Abstract:**

Day-old chick quality is an essential element for the overall profitability of the broiler productive cycle and has been associated with the growth performance and feed conversion rate. An effect on the development of the immune system was also reported, which could likely account for reduced susceptibility to infectious diseases and improved animal welfare parameters. Besides direct cost reduction, lower antimicrobial use and improved animal welfare are crucial in the directive of European Union legislation and are at the forefront of customer choices. Several factors contribute to determining the chick quality. Breeder flocks genetics, health, and management affect the egg features, quality, and bacterial load. However, hatchery practices play a pivotal role, since adequate hygiene and handling are fundamental in reducing egg contamination and cross-contamination. The presence of rotten eggs is often regarded as a major risk, since the internal bacterial load can contaminate the needle used for in-ovo vaccination, the nearby eggs, and the whole incubator/hatching room when broken. In the present multicentric study, representative of 40 hatcheries located in 11 European countries, a remarkable impact of the rotten egg percentage on the hatchery productive parameters, representative of the hatchability, embryo mortality, and level of contamination, was demonstrated. Efficient rotten egg removal and the application of appropriate detection and removal tools should thus provide remarkable benefits for hatchery performance and indirectly for downstream poultry production.

## 1. Introduction

Day-old chicken quality is of pivotal importance for the poultry meat-producing business. An improvement of this parameter will directly result in better performance in the farms, reduced antibiotic usage, decreased condemnation at slaughter, and improved product quality [1]. This improvement in chick quality has a significant impact on profitability for all elements in the value chain. For broiler farms, receiving better day-old chicks will result in a lower need for the usage of antibiotics during production and improved animal welfare parameters [2,3,4,5]. Especially within the European Union, antibiotic usage is today strongly discouraged and restricted [6].

In integrated poultry companies, chick quality is crucial to success, having implications in disease control and flock, feed, and facility management [7].

Apart from being demanded in integrated companies, chick quality provides also a competitive advantage for hatcheries in non-integrated companies and markets. Hatcheries that supply lower-quality chicks to their customers will be requested to compensate for the losses and, in repetitive cases, will lose customers. These economical and organizational laws have resulted in hatcheries focusing on the improvement and control of the quality of the day-old chickens leaving their production [8].

The first step in controlling the quality of chickens is assuring good egg quality and hygiene. This is even more crucial for hatcheries performing in-ovo vaccination [9]. The presence of rotten eggs is often regarded as a major risk, both for the internal bacterial load contaminating the needle used for in-ovo vaccination and for the potential of contaminating nearby eggs and the whole incubator/hatching room when broken [1,10]. Efficient rotten egg removal should thus provide remarkable benefits for hatchery performance and indirectly for downstream poultry production. However, the quantification of the impact of their removal can be challenging, since the detrimental effects can be confounded by many other factors, acting both at the breeder and hatchery level [11].

For this purpose, a multicentric study was performed on multiple hatcheries located in several European countries, receiving eggs from different breeder flocks of different ages to evaluate the impact of rotten egg removal on hatching parameters.

## 2. Materials and Methods

### 2.1. Data Collection

Data were obtained from broiler hatcheries where the Ceva Hatchery Immunization Control Keys (CHICK) program was implemented (Ceva Santé Animale, Libourne, France). This program involves teams of more than 175 specialists frequently auditing hatcheries in more than 40 countries worldwide to verify the vaccination process, secure the vaccination technology maintenance, train the hatchery personnel, and improve their performance.

During the audits, the different parameters are scored using a tablet-based application, and the results are stored in a dedicated database to be reported to hatchery management [12].

At the hatchery, data collection was performed as follows: The percentage of rotten eggs was recorded (1) automatically at transfer when using live candling by Laserlife^®^ live candling equipment (Ceva Ecat-iD Campus, Plouédern, France) or (2) manually at hatch by opening the non-hatched eggs. Thereafter, in all the flocks identified at transfer, the chickens were counted at hatching and their quality was evaluated. Particularly, from each flock 10 hatching baskets were randomly selected; the presence of living, dead, and culled chicks was scored; and the unhatched eggs were counted.

The following Key Parameters of Interest (KPI) were selected to measure the hatching performances:

A: Viable late embryos;
B: Culled, dead chicks, and late-hatched (wet chicks);
C: Hatch of viable embryos;
D: Embryo contamination;
E: Overall percentage of hatched eggs.

These KPI were selected since they represent different aspects of the overall hatching process and performance.

The percentage of potentially viable, late-mortality embryos (A) was assessed during the break-out after hatching of the non-hatched eggs.

The total number of dead embryos with an estimated embryo development equal or more than 17 days of age, the number of live-piping embryos, and the number of dead-piping embryos were recorded. From these categories, the total count was calculated and expressed as the percentage of the total initially set eggs. This number is representative of the eggs that were normally developed, but died during the last phase of incubation (in the hatcher room).

The total of culled chicks (i.e., chickens classified as fully hatched that are not viable over the first day of life), dead chicks, and late-hatched chicks (wet chicks) (B) was calculated by summing these three categories and calculating the percentage as a function of the number of initially set eggs.

The hatching of viable embryos (C) is defined as the number of chicks hatched at the moment of pull divided by the number of potentially valid, viable embryos at the moment of transfer. This number should be as close as possible to one hundred percent, indicating a successful transfer that did not negatively impact the hatch (for instance, by the contamination of the eggs/embryos). Therefore, this parameter allows one to evaluate the contribution of transfer, vaccination, and hatching to the overall performance, ruling out the impact of the incubation process.

The embryo contamination (D) was visually assessed by the presence of gas, mucous, fibrin, and specific smell. This parameter is expressed as the percentage of the total number of initially set eggs.

The overall hatchability (E) was included, since it has a major difference with parameter C. While parameter C is much “harder” and independent of external factors, D is more used in the field as its calculation is much easier and less demanding.

No experimental procedure has been performed in the present study, since all the data were obtained in the framework of normal hatchery activity. Therefore, no ethical committee approval was required according to the legislation of the countries where the study has been performed.

### 2.2. Statistical Analysis

The dataset was manually curated to identify and remove wrongly imputed data and biologically implausible outliers. All the records with at least one missing value in the variables of interest were removed from the analysis. The effect of the rotten egg percentage on the KPI—i.e., A, B, C, and D was evaluated independently using a linear mixed effect (LME) model, which allows the inclusion of both fixed and random effects. This approach was considered necessary, since the data were collected at different countries and hatcheries with a myriad of production practices. This intrinsic hierarchical structure leads to non-independence in the data set, preventing the use of standard regression models. Additionally, the hatchery management and handling practices at the country level may have an impact on the response variable; therefore, a hierarchical model was fitted, including the hatchery or the hatchery nested within the country as a random effect. Since the breeder flock age has a detrimental effect on the hatchability and is also correlated with rotten eggs, it was included in the models to account for its potential confounding effect. All the analyses were performed in R using the nlme package [13]. The normality and homoscedasticity of the residuals were graphically inspected for all the considered models. Several models were fitted, and the significance of each model improvement over the simpler one was assessed using the likelihood ratio test (LRT). Only the results of the best fitted-model were reported. The statistical significance threshold was set to *p* < 0.05 for all the performed tests.

## 3. Results

A total of 901 flock records (obtained from a minimum of 10 hatching baskets containing around 150 eggs—i.e., around 1500 eggs per record) originating from 40 hatcheries located in 11 European countries were included in the study. The breeder flock age ranged between 25 and 65 weeks (mean ± sd = 41.3 ± 9.36). More in detail, 13.65% were young (25–30 weeks), 36.61% were median (31–40 weeks), 30.73% were older (41–50 weeks), and 19.01% were old breeders (>51 weeks). Independently from the considered KPI, a significant improvement in the model fit was observed when the hatchery was included in the model as a random effect. Instead, no significant improvement was achieved if the country or hatchery, nested within the country, were added in the model. A significant association was proven between A (b = 0.627; standard error (SE) = 0.091; *p*-value < 0.001), C (b = −0.821; SE = 0.138; *p*-value < 0.001), D (b = −0.681; SE = 0.082; *p*-value = 0.002), and the overall hatchability (b = −3.43; SE = 0.68; *p*-value = < 0.001) and percentage of rotten eggs. On the contrary, no association could be identified with the B score (b = 0.015; SE = 0.058; *p*-value = 0.796) (Table 1). The effect of breeder age was significantly associated with all the considered KPIs, except for the A score (Table 1).

## 4. Discussion

The present study allowed us to consistently test and quantify the effect of rotten eggs on the hatchery KPI through the evaluation of 901 records performed in 40 hatcheries located in 11 European countries. A clear association between the percentage of rotten eggs and the decreased hatchery performances could be confirmed based on a large amount of collected data. Notably, a one-point increase in rotten eggs, on average, led to approximately 0.7% rise in contaminated embryos (D score) and a comparable increase in late viable embryo mortality (A score). This finding can be explained by the likely death of contaminated embryos at a late stage of development, causing hatching failure. Simultaneously, a ~1% decrease in hatchability (C score) of the eggs that pass the candling step was associated with a point increase in rotten eggs. An even higher reduction in the overall hatchability could be observed. These data confirm the significant consequences of inefficient egg selection in terms of hatchery profitability. A 100,000 Euro loss has been estimated for each 0.5% reduction in hatchability, assuming an average year processing of 60 million eggs and a 0.35 Euro selling price reduction for each day-old chick [14]. These financial losses should then be added to the increased treatment needs for the hatched chicks, higher feed conversion rate, slower growth, lower slaughter quality, etc., in the downstream production phase, which have been associated with poor day-old chicken quality [5,15,16]. Breeder age was previously linked with decreased performance, and the present study results confirmed this evidence [1,5,17]. Both egg quality and progressive increase in breeder housing contamination have been reported to be involved in this phenomenon [17]. Unfortunately, the hatchery control of the factors mentioned above is limited. It is thus especially relevant that, after accounting for breeder age, the residual effect of the rotten eggs was still significant. This variable is totally under hatchery control, and an effective egg selection can be achieved through adequate practices and the implementation of appropriate tools. Accordingly, the hatchery inclusion as a random effect led to a significant improvement in the model, testifying that the management practices at different hatcheries have a huge impact on the KPIs. The advancement in candling technologies which allows the effective and automatic exclusion of unsuitable eggs, including rotten ones, would be beneficial for the overall hatchery performance, especially when in-ovo vaccination is applied. Classic candling, which allows the removal of only clear and early dead embryos, is in this respect often not sufficient, especially not in the case of old breeder flocks.

In this context, new-generation candling techniques have recently become available and combining laser technology and thermal imaging to measure heat emission from the embryo is able to identify and remove rotten eggs, middle-dead embryos, late-dead embryos, and contaminated eggs at transfer. Therefore, their wider implementation could lead to overall improvements in hatchery performance and poultry health.

Surprisingly, the B score, including culled chicks, dead chicks, and late-hatched (wet) chicks, was not significantly associated with the rotten egg frequency. While this appears counter-intuitive, some issues have to be considered: (1) the subjectivity of this parameter, (2) the low number of cases (i.e., the number of culls, dead chicks, and late-hatched chicks is typically low, reducing the variability among groups; therefore, a much higher sample size could be required to detect a lower effect size).

A potential alternative, non-conflicting explanation could be related to the fact that, in a contaminated environment, a poorly developed chick will not hatch at all, while in non-contaminated settings a poorly developed chick will have a higher likelihood of hatching, but die thereafter or need to be culled. Therefore, a high percentage of rotten eggs could “paradoxically” reduce the number of culls, dead chicks, and late hatches, causing more precocious mortality. However, more focused studies will be necessary to confirm the above-mentioned hypothesis.

As a side note, such huge data acquisition and sharing were possible thanks to the development of a tablet-based software app and related data storage system. Further efforts should then be paid to continuously improve our data acquisition technologies, since they represent an invaluable starting point for the objective analysis and optimization of all the steps along the poultry production chain.

## 5. Conclusions

Overall, the present study demonstrated the remarkable effect of rotten eggs on hatchery performance. Their removal should represent a major aim of modern and efficient hatchery production. The better management and implementation of new-generation technologies could be of great benefit for the hatchery profitability; paying back the initial investment; and improving the overall downstream poultry health, welfare, and production.

## Figures and Tables

**Table 1:** Summary of the mixed effect linear regression parameters fitted for different Key Parameters of Interest (KPIs) and confounding variables; statistical significance is also reported. β1 = regression coefficient; A = viable late embryos; B = culled, dead chicks, and late-hatched chicks (wet chicks); C = hatch of viable embryos; D = embryo contamination; E = overall hatchability.

KPI	Variable	β1	Standard Error	*p*-Value
A: Viable late embryos	A	0.627	0.091	<0.001
	Breeder age	0.001	0.003	0.696
B: Cull, dead chicks, and late hatched	B	0.015	0.058	0.796
	Breeder age	0.017	0.002	<0.001
C: Hatch of viable embryos	C	−0.821	0.138	<0.001
	Breeder age	−0.027	0.005	<0.001
D: Embryo contamination	D	0.681	0.082	<0.001
	Breeder age	0.011	0.003	<0.001
E: Overall hatchability	E	−3.430	0.683	<0.001
	Breeder age	−0.186	0.027	<0.001
